# Vertebrate GAGA factor associated insulator elements demarcate homeotic genes in the *HOX* clusters

**DOI:** 10.1186/1756-8935-6-8

**Published:** 2013-04-22

**Authors:** Surabhi Srivastava, Deepika Puri, Hita Sony Garapati, Jyotsna Dhawan, Rakesh K Mishra

**Affiliations:** 1 Centre for Cellular and Molecular Biology, Council for Scientific and Industrial Research, Uppal Road, Hyderabad, 500007, India; 2 Institute for Stem Cell Biology and Regenerative Medicine, National Centre for Biological Sciences, Bangalore, 560065, India

**Keywords:** ChIP-on-chip, Chromatin domain boundary, Enhancer blocking, GAGA factor, Hox, Histone H3

## Abstract

**Background:**

*Hox* genes impart segment identity to body structures along the anterior-posterior axis and are crucial for the proper development of all organisms. Multiple regulatory elements, best defined in *Drosophila melanogaster*, ensure that *Hox* expression patterns follow the spatial and temporal colinearity reflected in their tight genomic organization. However, the precise mechanisms that regulate colinear patterns of *Hox* gene expression remain unclear, especially in higher vertebrates where it is not fully determined how the distinct activation domains of the tightly clustered *Hox* genes are defined independently of each other. Here, we report the identification of a large number of novel *cis*-elements at mammalian *Hox* clusters that can help in regulating their precise expression pattern.

**Results:**

We have identified DNA elements at all four murine *Hox* clusters that show poor association with histone H3 in chromatin immunoprecipitation (ChIP)-chip tiling arrays. The majority of these elements lie in the intergenic regions segregating adjacent *Hox* genes; we demonstrate that they possess efficient enhancer-blocking activity in mammalian cells. Further, we find that these histone-free intergenic regions bear GA repeat motifs and associate with the vertebrate homolog of the GAGA binding boundary factor. This suggests that they can act as GAGA factor-dependent chromatin boundaries that create independent domains, insulating each *Hox* gene from the influence of neighboring regulatory elements.

**Conclusions:**

Our results reveal a large number of potential regulatory elements throughout the murine *Hox* clusters. We further demarcate the precise location of several novel *cis*-elements bearing chromatin boundary activity that appear to segregate successive *Hox* genes. This reflects a pattern reminiscent of the organization of homeotic genes in *Drosophila*, where such regulatory elements have been characterized*.* Our findings thus provide new insights into the regulatory processes and evolutionarily conserved epigenetic mechanisms that control homeotic gene expression.

## Background

*Hox* genes specify segment identity during development: the unique combination of their expression pattern provides correct positional identity along the anterior-posterior (AP) body axis. The distinct organization of the *Hox* genes was first defined in *Drosophila melanogaster*[1] and such features as their structure, function, clustered organization, and spatial colinearity of expression (their order along the chromosome corresponds to their order of expression along the AP embryonic axis) are highly conserved in all metazoans [2]. Specific features of the vertebrate *Hox* complexes, however, are distinct from those seen in invertebrates. Through genome duplication events, vertebrates have at least four *Hox* clusters that are more organized, with all the genes transcribed in the same orientation and arranged more compactly than those in lower organisms [3]. The order of genes within the complexes, though, continues to be colinear, with their domain of expression along the AP axis: this shows remarkable evolutionary constraint to maintain the clustered organization and precise segment-specific expression patterns of the *Hox* genes. Their temporal order of activation, however, shows a difference; in *Drosophila*, the expression pattern of all the *Hox* genes is set simultaneously, while mammals show temporal colinearity by expressing their 3′ *Hox* genes first and then the more 5′ *Hox* genes sequentially from anterior to posterior during development [4,5]. These observations suggest that *Hox* gene regulation is highly dependent on genomic organization. It remains unclear how the colinear pattern of *Hox* gene expression in mammals is governed to provide discrete segment identity during development. In particular, it is not fully determined how the distinct activation domains of successive *Hox* genes are defined to maintain this spatial and temporal colinearity of expression. Mouse transgenes bearing regulatory regions of *Hox* genes faithfully recapitulate their expression domains [6,7], indicating that elements that define the domains of *Hox* gene expression are probably located in close proximity to the genes themselves, interspersed between coding regions within the conserved clusters. This arrangement may also allow enhancer sharing and promoter competition between the vertebrate *Hox* genes, providing an additional layer of regulatory control [8]. However, the complex interplay of *Hox* regulatory elements has not been clearly mapped in organisms other than *Drosophila*, where distinct regulatory domains control gene expression; most importantly, boundary elements flank each regulatory domain and define its limits in the bithorax complex [9]. Chromatin boundaries or insulator elements are implicated in regulating genome-wide chromatin-mediated effects and are crucial for blocking inappropriate gene expression to provide functional autonomy of chromatin domains [10,11]. Homeotic gene regulation is thus highly dependent on genomic organization and is maintained by progressive transcriptional activation from a repressed state governed by the trithorax (trxG) and polycomb (PcG) groups of proteins [12]. Epigenetic mechanisms therefore play a crucial role in facilitating chromatin reorganization in an ordered manner to provide accessibility control at *Hox* clusters [13].

Here, we have explored the chromatin regulatory features across all four murine *Hox* clusters using custom-designed high-resolution chromatin immunoprecipitation (ChIP)-on-chip tiling arrays to reveal the presence of novel histone H3-free regions as potential regulatory elements. We show that most of these histone-depleted regions are associated with the vertebrate GAGA factor. Further, almost all intergenic regions at the *Hox* clusters show the presence of such histone-free regions that bear significant enhancer-blocking activity in human cells. These findings establish an association of the GAGA factor at specific intergenic chromatin structures that act as regulatory elements in mammalian *Hox* clusters. The observation that some of these regions can function as chromatin boundaries indicates a conserved mechanism of *Hox* gene expression regulated by distinct chromatin domains of each gene.

## Results and discussion

### Identification of histone-free regions at murine *Hox* loci

To obtain a complete picture of the epigenetic organization of the four murine *Hox* clusters, we designed a ChIP-on-chip tiling array including all 39 *Hox* genes along with their intergenic as well as upstream and downstream flanking regions. Synchronized G0 cells from the mouse C2C12 cell line were used to obtain a homogenous assay system for chromatin analysis, minimizing possible effects of cell type or cell cycle distribution and potential variations in the epigenetic state. Chromatin immunoprecipitation (ChIP) experiments with histone H3 antibodies were then performed for hybridization to the custom-designed tiling arrays.

**Figure 1:**
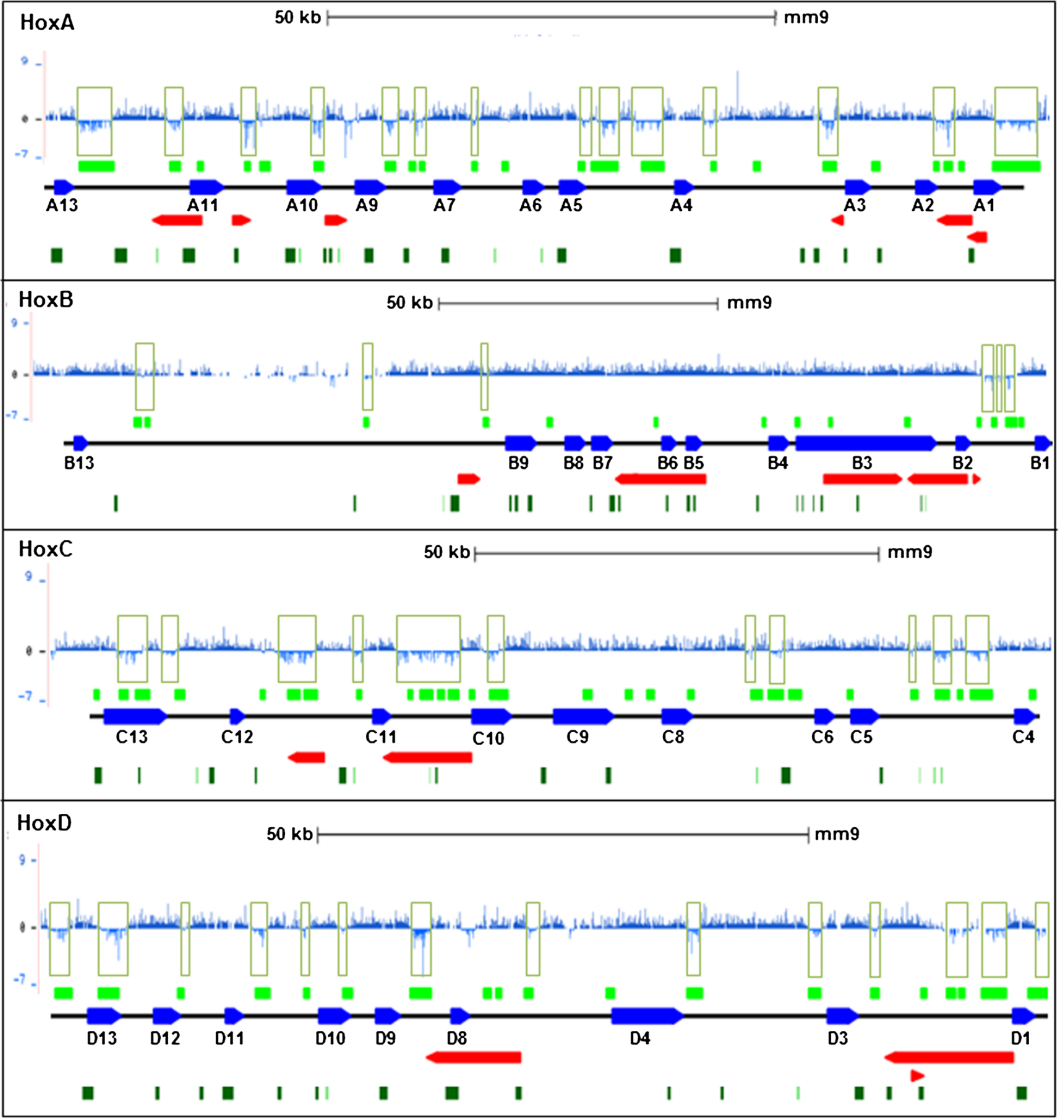
**ChIP-on-chip array reveals histone-free regions in the murine *Hox *clusters.** Screenshots from the histone H3 array data visualized using the UCSC genome browser [43] showing normalized log ratios (NLRs) of probes (*y* axis) at the *Hox* clusters. Large stretches of unenriched probes showing negative NLR values are highlighted by boxed regions. The genomic scale bar is indicated at the top. A map of all the HFRs (green boxes) within each cluster identified by bioinformatics analysis is presented at the bottom in the context of the genic regions (blue boxes with transcriptional orientation indicated; the large intronic regions of *Hoxa3* and *Hoxd3* genes have been omitted for clarity). Red boxes correspond to locations of noncoding transcripts (multiple transcripts mapping to same location are clubbed for clarity) and dark green boxes correspond to CpG islands.

Strikingly, ChIP-on-chip analysis revealed a consistent pattern of histone depletion at intergenic intervals across all arrays. The H3 ‘pan’ antibody against the carboxy terminus of histone H3 (an invariant region that escapes post-translational modifications) recognizes all forms of histone H3 and its modifications. We found that the binding of this antibody was depleted at many regions within the *Hox* clusters in a defined pattern, as outlined for 150 kb of each of the *Hox* clusters in Figure 1. These regions are likely to be devoid of histone H3, in contrast with the enriched regions in the pan histone H3 array that mark histone presence (Table 1), and were designated as histone-free regions (HFRs). Further, the HFRs showed consistent lack of enrichment with two additional modified histone antibodies specific for H3K4me3 and H3K27me3 (Additional file 1: Figure S1A). The four clusters displayed distinctly different patterns of peak enrichment with these two histone modification marks (Figure S1B) [14]. Antibody binding across the arrays was, however, used to identify the ‘unenriched regions’, marked by probes that showed consistently negative or poor enrichment values. Over 66% of the HFRs in the pan H3 array were found to be unenriched in all the three arrays (Additional file 1: Table S1). Thus, successive peaks of antibody enrichment were separated from each other by ‘troughs’ or ‘gaps’ that were not enriched for any of the antibodies. Most of the HFRs could be clearly visualized directly from the probe binding data by the presence of these large gaps in antibody enrichment (boxed regions in Figure 1 and Additional file 1: Figure S1A) suggesting an unequal histone organization throughout the Hox clusters, with the troughs of low histone H3 enrichment indicating regions of low occupancy.

**Table 1:** **Potential regulatory elements mapped as HFRs at the murine *Hox *clusters**

**HFR number**	**Name**	**Size**	**Context**	**HFR number**	**Name**	**Size**	**Context**
***HoxA***				***HoxC*continued**			
1	A_DOWN-1.1	5210	3′ end	8	C_12-11.1	510	Intergenic
2	A_1-2.1	510	NCT	9	C_12-11.2	1460	3′ end
3	A_1-2.2	860	NCT	10	C_12-11.3	1610	NCT
4	A_1-2.3	510	3′ end	11	C_12-11.4	510	Intergenic
5	A_2-3.1	810	Intergenic	12	C_11-10.1	510	NCT
6	A_3-4.1	1410	3′ end	13	C_11-10.2	1510	NCT
7	A_3-4.2	660	Intergenic	14	C_11-10.3	760	NCT
8	A_3-4.3	510	Intergenic	15	C_11-10.4	1260	NCT
9	A_4-5.1	2460	Intergenic	16	C_11-10.5	560	TSS
10	A_4-5.2	2920	Intergenic	17	C_10.1	2230	Genic
11	A_5.1	650	Genic	18	C_9.1	1010	Genic
12	A_6-7.1	610	Intergenic	19	C_9-8.1	760	Intergenic
13	A_6-7.2	510	Intergenic	20	C_9-8.2	850	Intergenic
14	A_7-9.1	510	Intergenic	21	C_8-6.1	710	3′ end
15	A_7-9.2	680	Intergenic	22	C_8-6.2	1370	Intergenic
16	A_7-9.3	1080	Intergenic	23	C_8-6.3	1810	Intergenic
17	A_9-10.1	960	3′ end	24	C_8-6.4	1510	Intergenic
18	A_10-11.1	1060	Intergenic	25	C_6-5.1	600	TSS
19	A_10-11.2	560	NCT	26	C_5-4.1	810	Intergenic
20	A_11.1	560	Genic	27	C_5-4.2	1660	Intergenic
21	A_11-13.1	1110	NCT	28	C_5-4.3	560	Intergenic
22	A_11-13.2	3810	Intergenic	29	C_5-4.4	2660	Intergenic
23	A.UP.1	510	Intergenic	30	C_4-DOWN.1	710	3′ end
24	A.UP.2	660	Intergenic	31	C_DOWN.1	660	Intergenic
25	A.UP.3	560	Intergenic	32	C_DOWN.2	1580	Intergenic
***HoxB***				***HoxD***			
1	B_13-9.1	1160	Intergenic	1	D_UP.13	860	Intergenic
2	B_13-9.2	760	Intergenic	2	D_UP.14	1660	Intergenic
3	B_13-9.3	720	Intergenic	3	D_13.1	2030	Genic
4	B_13-9.4	810	Intergenic	4	D_12-11.1	560	3′ end
5	B_9-8.1	800	Intergenic	5	D_11-10.1	1410	Intergenic
6	B_7-6.1	510	NCT	6	D_11-10.2	510	Intergenic
7	B_5-4.1	510	TSS	7	D_10-9.1	910	3′ end
8	B_4-3.1	710	TSS	8	D_9-8.1	2080	3′ end
9	B_3.1	510	Genic	9	D_8-4.1	710	NCT
10	B_3.2	820	Genic	10	D_8-4.2	510	NCT
11	B_2-1.1	560	3′ end	11	D_8-4.3	810	TSS
12	B_2-1.2	760	Intergenic	12	D_8-4.4	760	TSS
13	B_2-1.3	1880	Intergenic	13	D_4-3.1	1510	Intergenic
14	B_2-1.4	690	Intergenic	14	D_4-3.2	1160	TSS
***HoxC***				15	D_3-1.1	760	Intergenic
1	C_UP.26	1020	Intergenic	16	D_3-1.2	510	3′ end
2	C_UP.27	2260	Intergenic	17	D_3-1.3	840	NCT
3	C_UP.28	510	Intergenic	18	D_3-1.4	590	NCT
4	C_UP.29	530	TSS	19	D_3-1.5	2470	NCT
5	C_13.1	1010	Genic	20	D_1-DOWN.1	1360	3′ end
6	C_13.2	1710	Genic	21	D_DOWN.1	3680	Intergenic
7	C_13-12.1	1110	Intergenic	22	D_DOWN.2	1080	Intergenic

93 HFRs at the *Hox* clusters showing low histone occupancy, as identified from the histone H3 ChIP-on-chip array, are listed along with their contextual location. Nomenclature of the HFR indicates the flanking *Hox* genes. TSS = HFRs within 1 kb of TSS; 3′end = HFRs at 3′ end of genes; NCT = HFRs within noncoding transcribed regions; Genic = HFRs within *Hox* genes. Regions located outside the clusters (within 10 kb upstream and downstream) have been associated with the closest *Hox* gene.

Nucleosome organization governs the accessibility of chromatin to regulatory complexes, increasing the binding of nonhistone proteins to target sites. Genome-wide chromatin studies have established the presence of regions with altered nucleosome occupancy, which often coincide with known regulatory elements, such as gene promoters, enhancers and transcription start and termination sites [15]. The distribution of core histone H3 has been used previously as a readout of nucleosome distribution in yeast [16] and other organisms, including mouse and human [17,18]. To identify all genomic regions associated with poor histone H3 presence in the context of their location within our arrays, we used a bioinformatics approach, considering the individual histone enrichment at neighboring probes at high resolution to delineate the extent of all possible HFRs at the four clusters (see Methods). Using custom scripts, we could identify many more regions from our ChIP-on-chip array that showed poor enrichment and were likely to be part of HFRs at these clusters. The location of all the HFRs thus identified is indicated in Table 1 and in the HFR maps provided below each cluster in the context of genic regions in Figure 1 and Additional file 1: Figure S1A (green boxes). The HFRs were most frequent across the *HoxA*, *HoxC*, and *HoxD* clusters while the *HoxB* cluster showed the lowest occurrence of such regions. The *HoxB* cluster is different from the other *Hox* clusters in that the *Hoxb13* gene is separated from the rest of the cluster by ~70 kb of DNA, in contrast with the other homeotic genes, which are closely spaced in all four clusters. Although the *HoxB* cluster maintains colinear expression of all its genes along the A-P body axis, the distant location of *Hoxb13* is believed to contribute to its loss of expression along the secondary body axes, presumably owing to the absence of *cis*-regulatory features in the large intervening region, which also contains highly repetitive DNA sequences [19]. This unique feature of the *HoxB* cluster could explain the absence of HFRs in this part of the complex. The reason behind the low frequency of HFRs in the cluster as a whole is not clear, although it may suggest a relatively low regulatory complexity for this cluster.

Interestingly, we observed that many of the HFRs were positioned intergenically. Altogether, 93 HFRs were identified by bioinformatics analysis at the Hox clusters (including the 10 kb flanking upstream and downstream regions) of which only 9 were genic (Table 1). Of the intergenic HFRs, 9 were within 1 kb around a gene start and could be considered to overlap with putative promoter regions while 11 were located at the 3′ end of *Hox* coding regions. A large number of the intergenic HFRs (64 HFRs) were located at intermediate positions between successive *Hox* genes or downstream to them. Apart from the *Hox* coding regions, we also checked the positions of intergenic noncoding transcripts as well as all CpG islands known at the *Hox* clusters. While 16 of the intergenic HFRs were found within noncoding transcribed regions, the remainder showed no overlap with any of these known intergenic features (Figure 1), suggesting that many of these HFRs probably represent sites for other chromatin regulatory activities. In *Drosophila*, it has been shown that the binding of promoter-proximal stalling factors at inactive *Hox* genes helps organize insulator loop domains [20]. The finding of histone-depleted regions around mammalian *Hox* genes, therefore, suggests a potential for the binding of regulatory proteins at these sites to bring about such chromatin reorganization activities.

### Histone-free regions at the *Hox* clusters function as enhancer blockers

Histone depletion is a feature of genomic sites involved in regulatory activities as it allows for binding of nonhistone target proteins. Given the distinct spatiotemporal activation of mammalian *Hox* genes during development, we hypothesized that the intergenic HFRs identified in our array represent sites for regulatory elements that could help define functional domains and, thereby, the precise expression patterns of these genes. *Hox* genes are organized in a cluster, yet they are uniquely regulated in a spatially and temporally restricted manner such that each gene can contribute to a unique segment identity. This necessitates the presence of functionally independent domains for each gene within the cluster defined by chromatin domain boundaries that can ensure unique expression patterns in each segment. In vertebrates, such elements have not yet been clearly defined at the *Hox* clusters.

**Figure 2:**
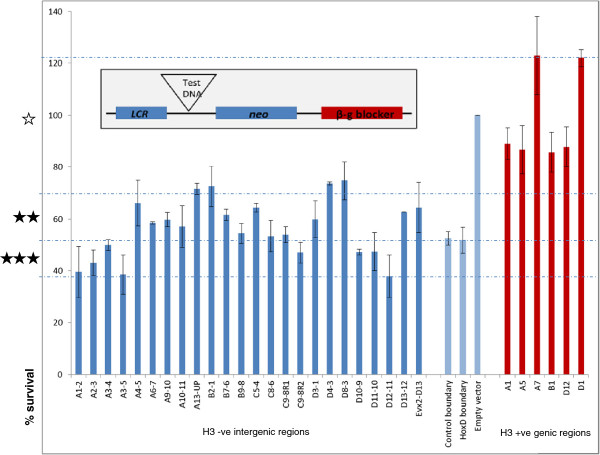
**Intergenic HFRs function as enhancer blockers.** The human erythroleukemic cell line K562 was stably transfected with different constructs carrying test fragments from the Hox clusters, including intergenic H3-free target regions (dark blue bars) and H3-enriched genic controls (red bars). The inset shows the map of the vector used for the boundary assay. Empty vector negative control and two known boundary constructs (positive controls) carrying the chicken β-globin boundary (control boundary) and the previously characterized *Hoxd13*-*Evx2* intergenic boundary (*HoxD* boundary) are indicated with light blue bars. The ratio of survival of average number of colonies with each construct normalized to the empty vector is shown (%) on the *y* axis. Dotted lines indicate levels of boundary activity observed based on the positive and negative controls, with the unfilled star marking the region of negligible activity comparable to negative control and filled stars indicating regions encompassing increasingly efficient enhancer-blocking activity comparable to the positive controls. Results shown are the mean ± standard error from two independent biological replicate experiments, each performed in triplicate.

To determine whether any of the newly identified HFRs marked potential boundary elements at the murine *Hox* clusters, we decided to test their enhancer-blocking ability. We selected candidate HFRs that were located in intergenic regions and showed no overlap with any of the known regulatory features at the clusters as described. The average size of the test elements was about 1 kb; all of the test elements showed negligible enrichment with the histone H3 antibody. The HFRs from all four clusters were tested for enhancer-blocking activity in a human cell line using the number of neomycin resistant colonies formed as a readout of enhancer activity. A boundary element cloned between the locus control region (LCR) enhancer and the *γ-neo* gene providing antibiotic resistance blocks promoter activation (Figure 2 inset), thereby rendering the cells sensitive to G418 and decreasing the number of colonies obtained in this assay. Earlier reports have established that the presence of lambda insert between LCR and *γ-neo* does not have any effect on the expression of the neomycin resistance gene, ruling out any distance effect [21]. We used the vector alone as a negative control to indicate 100% survival of colonies in the absence of any blocking of the *γ-neo* gene and the well-known chicken β-globin boundary [21] as a positive control against which to compare the number of colonies obtained with the test regions from the *Hox* clusters. We tested 24 intergenic HFRs and, as a control for specificity, we also included 6 regions (from within *Hox* gene bodies) that showed high enrichment with the core histone H3 antibody.

Strikingly, we observed that there was a significant decrease (*P* < 0.0001) in the number of G-418 resistant colonies when cells were transfected with the target HFRs (Figure 2). The control chicken β-globin boundary element showed 53% survival of colonies in the assay. A fragment from the *Hoxd13-Evx2* intergenic region that we had previously characterized as a potential boundary element [22] was also as efficient in enhancer blocking as the chicken β-globin element in our assay (52% survival). Over this background, we found that eight of the HFRs tested showed substantially fewer colonies (only 38% to 50% survival) than those obtained with both control boundaries in the same assay, indicating extremely efficient enhancer-blocking activity associated with these intergenic regions and suggesting their potential to act as strong boundary elements. Further, 12 more of the tested HFRs also displayed enhancer-blocking activity comparable to the control boundaries (50% to 65% survival). Only 4 regions out of the 24 intergenic fragments tested showed survival >65% (but still <75%). Thus, 20/24 intergenic HFRs tested behaved as efficient enhancer blockers in this assay, suggesting that these histone-free regions could act as boundary elements and demonstrating a correlation between the presence of HFRs and their role in demarcating *Hox* gene domains. In contrast, in the case of the six H3-enriched fragments derived from the *Hox* genic regions, consistently high survival of colonies could be observed (85% to 123% survival; red bars in Figure 2), similar to or greater than that seen with the vector control, indicating an absence of enhancer-blocking potential. Taken together, these results clearly show that the intergenic HFRs from the murine *Hox* clusters selectively block the LCR enhancer from acting on *γ-neo* reporter gene, indicating that these sequences can function as boundary elements.

The disruption of histones exposes *cis*-regulatory elements and their binding motifs in contrast with the surrounding packed DNA. Boundary elements can thus be expected to be associated with a modified chromatin state and indeed, the scs and scs′ elements at the Hsp70 locus in *Drosophila* were originally characterized by their specialized chromatin structure [23]. Reduced nucleosome occupancy corresponding to histone replacement has since been reported at functional boundaries within the *Drosophila* bithorax complex and the fact that these are correlated with PcG and trxG protein binding suggests an increased accessibility of the PREs (polycomb response elements) and boundary elements [24]. In this context, our results suggest that mammalian *Hox* clusters also bear such regulatory elements and this study provides a large-scale identification of such potential elements marked by their modified histone occupancy pattern. Not all the elements tested displayed strong boundary potential and it is possible that the enhancer blocking observed in some cases may be the manifestation of some other activity in their native context, as there are many ways that enhancer-promoter interactions can be disrupted [10,25]. Based on the genomic context and the fact that some of the HFRs bear strong enhancer-blocking activity, we suggest the presence of chromatin domain boundaries at the intergenic locations identified here. This finding indicates a conserved mechanism to control the initiation and maintenance of collinear *Hox* gene expression in mammals.

### Intergenic HFRs at the *Hox* clusters are associated with the vertebrate GAGA binding factor

**Figure 3:**
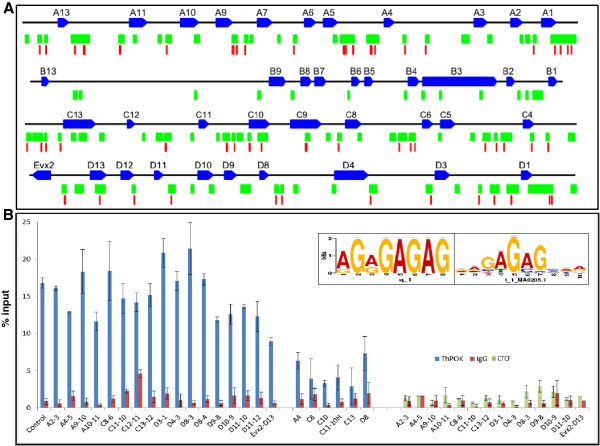
**Vertebrate GAGA factor (*Th-POK*) is associated with intergenic HFRs.** Sequence analysis of HFRs from *Hox* clusters reveals the presence of multiple GAF binding sites. (**A**) Schematic map (drawn to scale) depicting the location of all the GAGA motifs (vertical red lines). A few motifs appearing close together are merged. Blue boxes denote the *Hox* genes with transcriptional orientation (the large intronic regions of *Hoxa3* and *Hoxd3* genes have been omitted for clarity); green boxes indicate the HFRs. (**B**) Binding of *Th-POK* (vertebrate GAF) and CTCF across the *Hox* clusters was assessed by real time ChIP-qPCR assays using a panel of primers designed at the HFRs as well as some genic regions enriched for histone H3. The known *Th-POK* binding region at the *Evx2-Hoxd13* boundary [22] was used as a reference (control). ChIP with *Th-POK* antibody (left) showed robust binding measured as percentage of input at the control and intergenic HFRs (blue bars) enriched above the background level of the nonspecific immunoglobulin G (IgG, red bars). Genic regions did not show such a high enrichment profile (middle). ChIP with the CTCF antibody (right) did not show any enrichment over the background at the HFRs. Results represent mean ± standard error from three independent experiments with each qPCR assay performed in triplicate. Inset depicts the motif discovered from the HFRs (left) and matched to the GAGA binding site (right) by MEME analysis.

The observation that a large number of the HFRs act as enhancer blockers in the *in vitro* colony formation assay suggests their possible association with key regulatory factors at the murine *Hox* loci. We therefore subjected all the identified HFRs to sequence analysis and found multiple sites for the GAGA binding factor (GAF in *Drosophila*) at the histone-free regions, with the GAGAG motif appearing as the most frequent significant hit with a motif *P* < 0.0005 (Figure 3 inset). In all, 44 intergenic HFR sequences were found to contain putative GAF binding sites, many with multiple instances or repetitions of the GAGAG motif (Figure 3A). A total of 113 GAF motifs could be identified throughout the HFRs at the *HoxA*, *HoxC*, and *HoxD* clusters, including 10 kb upstream and downstream flanking regions (Additional file 1: Table S2). Interestingly, the *HoxB* cluster showed a lack of association of its few HFRs with the predicted GAF motif. This suggests the existence of other non-GAF-dependent mechanisms, especially in the regulation of the *HoxB* cluster. Alterations in histone modifications and chromatin decondensation followed by a stepwise looping out of the active genes are implicated in temporal *HoxB* gene expression [26] but the factors involved in this reorganization are unknown.

GAF is a versatile DNA binding factor, known to be associated with nuclease hypersensitive sites, that binds to GAGAG stretches to perform complex multifunctional roles in genome regulation [27]. Although identified as a trxG component in the context of *Hox* regulation at active chromatin within regulatory elements in *Drosophila*[28], GAF was subsequently shown to be associated with enhancer-blocking activity as well and GAF-dependent boundaries mediate the activity of multiple homeotic enhancers in *Drosophila*[29,30]. To confirm whether the intergenic HFR sequences were associated with GAGA factor in mouse cells, we then performed ChIP-qPCR analysis using an antibody directed against *Th-POK* (the vertebrate homolog of dGAF). Enrichment was assayed using multiple primers designed across 18 HFRs, including 13 that had shown high enhancer-blocking activity, as described in Figure 2. The known *Th-POK* binding boundary region between *Evx2-Hoxd13* characterized previously ([22]; control in Figure 3B) was used as a reference and showed robust association with the *Th-POK* antibody, much greater than that observed for the IgG control in the ChIP assay. We found that most of the HFRs that had shown enhancer-blocking activity were also similarly highly enriched for *Th-POK* binding (Figure 3B). Five additional intergenic HFRs that had not been tested for enhancer-blocking activity but showed low histone occupancy also showed significant *Th-POK* association, indicating that these are also likely to bear boundary potential. The high degree of enrichment indicates robust association with GAGA factor at these regions, correlating well with the presence of multiple GAF binding sites at the HFRs. On the other hand, six regions with high histone occupancy in the histone H3 array (H3-enriched or H3-positive regions), positioned within the Hox gene bodies, did not show similarly robust association with the *Th-POK* antibody (Figure 3B, middle).

CTCF (CCCTC binding factor) is the best studied vertebrate boundary factor (a large number of CTCF binding sites have been found in the mammalian genome [31,32]) and also plays many roles in genome organization and regulation. At the *HoxA* cluster, CTCF has been shown to maintain higher-order architecture via barrier activity, and CTCF in conjunction with cohesin organizes chromosome looping to segregate inactive *HoxA* gene domains [33]. We therefore decided to check for CTCF binding site association with the HFRs at the *Hox* clusters. However, we could not find any evidence of preferential association of CTCF motifs within the intergenic HFRs by sequence analysis, as seen for the GAF binding sites. A comparison with CTCF binding sites identified by the ENCODE project also showed minimal overlap with HFRs (Additional file 1: Figure S2). We further checked for CTCF binding at 14 intergenic HFRs by ChIP-qPCR analysis. In contrast with the clear association of *Th-POK*, CTCF was not significantly enriched in comparison with the control IgG antibody at most of the boundary or intergenic HFRs tested (Figure 3B, right). Thus, although CTCF is known to be the commonly associated vertebrate insulator factor, it is unlikely to be the mediator of the regulatory activity associated with these newly described HFRs in the *Hox* loci. These results are consistent with recent work demonstrating that CTCF does not act as a boundary element at the *HoxD* cluster [34]. CTCF can organize genome regulation through diverse activities, such as transcriptional activation and repression [35]. It is possible that CTCF binds to other sites not defined by low levels of H3-enrichment as assayed in this study, where it may play direct roles in the transcriptional regulation and chromatin compaction of the *Hox* genes [36].

### HFRs overlap with the sites of DNaseI hypersensitivity at the *Hox* clusters

The association of HFRs observed here appears to be specifically with the vertebrate GAGA factor and could possibly be the outcome of the nucleosome-reorganizing potential of GAF and not solely a feature of boundary activity. In *Drosophila*, many *cis*-regulatory elements including boundaries within the homeotic complex are bound by GAF, and evidence suggests that altering the nucleosome occupancy might be a feature of such binding [37]. To determine whether HFRs marked sites of histone displacement, we checked for the presence of DNaseI hypersensitive (HS) sites using published data from ENCODE. A large number of the total HFRs overlap with a range of HS sites identified in mesodermal tissue and skeletal muscle (tissue of origin) as well as embryonic stem cells (Additional file 1: Figure S3). 82% of the HFRs identified in this work mapped to an HS site in at least one of the cell types examined while 27 HFRs showed an overlap with HS site consistently across all the cells (Additional file 1: Table S3). DNaseI hypersensitivity serves as a measure of chromatin accessibility and is therefore a reliable marker for sites of histone disruption and associated regulatory activity. The presence of an HS site could be a dynamic feature when it marks regulation triggered by a transcriptional event. Consistent association with nuclease hypersensitivity across cell types suggests higher-order chromatin structure, as in the case of boundary elements, such as scs and scs′ in *Drosophila*, which remain associated with HS sites regardless of a heat-shock event triggering the activation of the locus [23].

We hypothesize that the vertebrate GAF-associated intergenic HFRs described here may be important in maintaining the local chromatin conformation of *Hox* genes. It remains to be determined if the histone depletion creates accessibility at these sites for enhancer-blocking function or if the GAF binding elements actively serve as anchors for directing the basic chromatin structure by histone disruption at the *Hox* complexes. dGAF plays a key role in directing chromatin architecture and driving nucleosome reorganization by recruiting remodeling complexes; its mutation has been shown to effect both histone replacement and associated boundary function at *Hox* complexes in *Drosophila*[38]. The presence of multiple GAF sites in the intergenic HFRs suggests the possibility of the vertebrate GAGA factor directing nucleosome reorganization and allowing binding of regulatory proteins at mammalian *Hox* clusters. Since HFRs can provide sites for a multitude of target proteins and GAF can bind to various proteins to form regulatory complexes, looking for interacting partners at these sites will help identify other regulatory features at the HFRs. Taken together, our findings suggest a correlation between GAGA factor association at the histone-depleted regions and chromatin accessibility, providing functional significance to the presence of HFRs in the intergenic regions of mammalian *Hox* clusters.

## Conclusions

This study used an epigenetic approach to identify a novel set of DNA elements at murine *Hox* clusters potentially involved in higher-order chromatin regulatory mechanisms. In *Drosophila*, multiple elements, such as enhancers, insulators, and PREs, form regulatory units that determine and maintain the segment-specific expression pattern of the associated homeotic genes. In mammals, the regulatory elements driving colinear activation and maintenance of *Hox* expression have not been delineated. In this context, GAGA factor associated histone-depleted regions identified in the intergenic stretches between murine *Hox* genes suggest sites of potential regulatory function that could help demarcate their domains of expression. We have previously characterized a GA-rich intergenic tract between the *Evx2* and *Hoxd13* genes conserved among mouse, human, and zebrafish and demonstrated its functional conservation as an enhancer blocker in both transgenic flies and cultured human cells [22]. The region encompassing the GA site has also been shown to act as a chromatin domain boundary element at the murine *HoxD* locus [39]; we have further shown that specifically mutating the GAGA binding sequence abolishes its insulator function [22]. We have previously established *Th-POK* as the vertebrate homolog of dGAF and indicated its potential to bind and regulate *Hox* clusters [40]. In this work, we have described a marked association of HFRs across the murine *Hox* clusters with GAF recognition sites and *Th-POK* binding. These results indicate a strong correlation of HFRs associated with *Th-POK* binding as a general feature of mammalian *Hox* gene domain regulation and provide mechanistic insights into the possible role of the mammalian GAGA factor in nucleosome reorganization at the *Hox* clusters, thereby setting the stage for the binding of regulatory proteins to organize chromatin regulatory activities, including boundaries.

Finally, sequence comparison might not directly identify homologous regulatory elements of the *Hox* genes across species but delineating the conserved organizational features of the *Hox* clusters can offer remarkable insights into their regulation. In this study, we have been able to identify over 90 such novel regions in the *Hox* clusters as HFRs and demonstrate the potential of many of the intergenic HFRs to function as chromatin regulatory elements. Our work indicates a genome-wide association for such structures and demonstrates that a lack of enrichment of histone H3 at specific genomic sites in ChIP-on-chip binding assays can serve as a marker for the identification of novel regulatory elements in a context-specific manner.

## Methods

### Cell culture

C2C12 skeletal muscle myoblasts were cultured as described previously [41]. Undifferentiated myoblasts were maintained in growth medium containing DMEM with 20% FBS and synchronized by subjecting subconfluent cells to suspension culture at a density of 10^5^ cells/ml in DMEM containing 1.3% methyl cellulose, 20% FBS, 10 mM HEPES, and 1 × Penicillin-Streptomycin. Suspended cells were harvested after 48 h, during which time 98% of cells were arrested at the quiescent G0 stage [42], by dilution with 1 × PBS and centrifugation at 2500 rpm for 30 min at room temperature. Cells were washed well with 1 × PBS at room temperature to remove all traces of methyl cellulose and pelleted for chromatin isolation.

### Chromatin immunoprecipitation

Chromatin was crosslinked and isolated from 10^7^ synchronized cells. Cells were harvested as described and fixed using 1% formaldehyde (Fisher Scientific) in growth medium for 10 minutes at 37°C and quenched with 0.125 M Glycine (Sigma). Fixed cells were washed well with 1 × PBS containing protease inhibitors at 4°C and resuspended in 2 ml lysis buffer supplemented with PMSF, DTT, and protease inhibitor cocktail (Roche). Following 15 min incubation on ice, the sample was sonicated using Bioruptor (Diagnode) to obtain fragments of average size 200 bp to 600 bp. The sonicated chromatin was divided into 200 μl aliquots for chromatin immunoprecipitation (ChIP) using the ChIP assay kit (Upstate, #17–295) according to the manufacturer’s protocol. ChIP was performed on precleared chromatin from 10^6^ cells using rabbit polyclonal antibodies against core histone H3 (Abcam, #ab1791), H3K4me3 (Millipore, #07–473), H3K27me3 (Millipore, #07–449), *Th-POK* (Abcam, #ab20985), or CTCF (Abcam, #ab70303). Unenriched input fraction was retained as control in each case. ChIP and input samples were subjected to array hybridization or real time qPCR assays to determine enrichment.

### Tiling array design and hybridization

A custom mouse genome microarray chip representing 1.1 Mb of the mouse genome consisting of the four *Hox* clusters was designed with 60-mer probes (approximately 16,200 probes) tiled continuously with a 10 bp overlap between consecutive probes for high resolution (Agilent Technologies). Repeat masking was used to avoid repeat sequences and other probes with cross hybridizing potential. Dual-color microarray hybridization experiments were carried out according to the manufacturer’s protocol. Briefly, samples recovered from the enriched fraction and unenriched whole cell extracts (input DNA) from two parallel experiments were independently pooled to obtain optimal yields for direct array hybridization, thus minimizing bias introduced during ligation-mediated PCR amplification of DNA fragments. The pooled enriched and input fractions were directly labeled with Cy5 and Cy3 dyes, respectively, using an Agilent Genomic DNA labeling kit (5190–0449) and hybridized onto the mouse custom arrays at 65°C for 40 h followed by washing. Scanned microarrays were submitted for background subtraction and data extraction to Agilent’s Feature Extraction. Normalized enrichment values for the probes were identified using DNA Analytics software. Probe data were normalized using default blanks subtraction and intra-array dye-bias median normalization and *P* values were assigned to groups of neighboring probes using the Whitehead error model.

The ChIP-on-chip signals were normalized with input DNA signals hybridized to the same arrays and the NLRs of the probes thus obtained were visualized on the Mouse NCBI37/mm9 Assembly in the UCSC genome browser [43]. Additional file 2 includes the NLR values of all the probes from the Hox clusters. The tiling array data have been submitted to the GEO database ([44]; GEO:GSE42941).

### Bioinformatic analysis

For data analysis and definition of all the histone unenriched gaps (HFRs), NLR values of the probes within the *Hox* clusters from the histone H3 tiling array were used to identify unenriched genomic stretches using an in-house PERL script. The extent of each target region was determined based on a defined cut-off for the number of consecutive probes that showed a negative NLR value with the histone H3 antibody. Briefly, the probe dataset from each cluster was divided into groups of five contiguous probes and each was classified as a ‘low enrichment’ group if at least three probes in the group had an NLR <0. Continuous blocks of low enrichment groups were extended until two or more consecutive high enrichment groups were encountered or if a genomic distance >200 bp was encountered between successive probes. These poorly enriched genomic stretches were classified as histone H3-free regions or HFRs and subjected to further analysis for ChIP-qPCR (Primers are provided in Additional file 1: Table S4) and enhancer-blocking assays.

Genomic sequences of HFR stretches from each of the clusters were extracted from the mouse reference assembly MGSCv37-C57BL/6J of NCBI build 37.2 and subjected to motif search analysis using the MEME Suite of tools [45]. The HFRs were mapped in the context of the murine *Hox* genes and all noncoding transcripts (downloaded from the Tromer database) using Geneious [46].

### Enhancer-blocking assays

HFRs and H3-enriched genic control regions selected from the *Hox* clusters were amplified as test fragments from mouse genomic DNA using primers designed with Xho 1 restriction sites at their 5′ ends (Additional file 1: Table S5). The PCR products were then purified by gel extraction and cloned into pCR-Blunt II Topo vector provided in the zero blunt TOPO PCR cloning kit (Invitrogen, #K2800-20) according to the manufacturers’ instructions. Following excision from the TOPO vector using Xho1 restriction enzyme, the test fragments were cloned into the pJC5-4-Xho boundary assay vector, which is an altered form of the pGEM-4Z vector containing the mouse 5-HS2 globin LCR as enhancer, a neomycin resistance gene as reporter and a chicken β-globin insulator. The test fragments were cloned between the enhancer and the reporter and all the constructs, including intergenic HFR target regions and some H3-enriched genic controls, were transfected into human K562 erythroleukemia cells, as described previously [22]. Colony assays were carried out according to standard procedures [21]. Relative numbers of surviving colonies in G418 medium for each construct were calculated relative to the empty vector-transfected colonies. Results were determined from two independent biological replicate assays for all the constructs, each carried out in triplicate. The statistical significance of the difference in the number of colonies obtained with the HFR target region constructs compared to the vector control was calculated using the unpaired Student’s *t* test.

## Abbreviations

AP: anterior-posterior; ChIP: chromatin immunoprecipitation; CTCF: CCCTC binding factor; DMEM: Dulbecco’s modified Eagle’s medium; DTT: dithiothreitol; FBS: fetal bovine serum; GAF: GAGA binding factor; HFR: histone-free region; HS: hypersensitive; LCR: locus control region; NLR: normalized log ratio; PBS: phosphate buffered saline; PcG: polycomb; PCR: polymerase chain reaction; PMSF: phenylmethanesulfonylfluoride; PRE: polycomb response elements; qPCR: quantitative polymerase chain reaction; TrxG: trithorax.

## Competing interests

The authors declare that they have no competing interests.

## Authors’ contributions

SS, RKM, and JD designed the study and wrote the manuscript. SS designed the tiling array and performed ChIP-on-chip analysis and ChIP-qPCR experiments. DP cloned test constructs and performed enhancer-blocking assays with SS. HSG carried out the bioinformatics analysis. All authors read and approved the final manuscript.

## Supplementary Material

Additional file 1: Figure S1 HFRs across all arrays using histone H3 antibodies. Screenshots from the tiling array data obtained using pan histone H3 (blue track), H3K4me3 (pink track) and H3K27me3 (green track) antibodies, visualized using the UCSC genome browser showing normalized log ratio (NLR) values of probes (*y* axis) at the Hox clusters (*x* axis). (**A**) Large stretches of histone H3 unenriched probes showing negative NLR values across all the arrays are highlighted by boxed regions. Map of all the HFRs (green boxes) further identified by bioinformatics analysis of the probe binding data is presented at the bottom for each cluster. (**B**) *H3K4me3* and *H3K27me3* peaks called in the custom tiling arrays show that the *HoxB* and *HoxD* clusters are highly enriched for the H3K27me3 mark in G0 cells, as reported previously for proliferating myoblasts and myotubes [14]; genes in the *HoxA* and *HoxC* clusters do not share this feature but show some association with *H3K4me3*. These trends are comparable with those seen in the ChIP-seq data from ENCODE using C2C12 cells (brown tracks). Genomic scale bar is indicated at the top. **Figure S2.** Map of GAGA motifs and CTCF sites across *Hox* clusters. Schematic map (drawn to scale) depicting the location of all the GAGA motifs identified by sequence analysis as well as the CTCF sites identified by ChIP-seq in C2C12 cells from the mouse ENCODE project as obtained from the UCSC genome browser (Transcription Factor Binding Sites by ChIP-seq from ENCODE/Caltech). Green boxes indicate HFRs while red bars mark GAGA motifs and grey bars mark CTCF sites. Blue boxes denote the *Hox* genes with transcriptional orientation; the large intronic regions of *Hoxa3* and *Hoxd3* genes have been omitted for clarity. **Figure S3.** DNaseI HS peaks in context of HFRs at the *Hox* clusters. Schematic map (drawn to scale) depicting the location of all the peaks of DNaseI hypersensitivity identified in skeletal muscle (blue track), mesoderm (black track) and embryonic stem cells (maroon track) from the mouse ENCODE project (DNaseI Hypersensitivity by Digital DNaseI from ENCODE/University of Washington). Green boxes indicate the HFRs while blue boxes denote the *Hox* genes with transcriptional orientation; the large intronic regions of *Hoxa3* and *Hoxd3* genes have been omitted for clarity. **Table S1.** Classification of HFRs identified in the three histone arrays. **Table S2.** List of GAGA factor binding motifs at the *Hox* clusters. **Table S3.** HFRs overlapping with DNaseI HS sites. **Table S4.** List of primers for ChIP-qPCR assays. **Table S5.** List of primers for cloning test fragments for boundary assays. Click here for file

Additional file 2 **Normalized log ratio values for probes at *Hox *clusters.** Click here for file

## References

[B1] Lewis EB A gene complex controlling segmentation in *Drosophila* Nature 1978 276 565 570 10.1038/276565a0 103000

[B2] Graham A Papalopulu N Krumlauf R The murine and *Drosophila* homeobox gene complexes have common features of organization and expression Cell 1989 57 367 378 10.1016/0092-8674(89)90912-4 2566383

[B3] Krumlauf R *Hox* genes in vertebrate development Cell 1994 78 191 201 10.1016/0092-8674(94)90290-9 7913880

[B4] Izpisua-Belmonte JC Falkenstein H Dolle P Renucci A Duboule D Murine genes related to the *Drosophila AbdB* homeotic genes are sequentially expressed during development of the posterior part of the body EMBO J 1991 10 2279 2289 1676674 10.1002/j.1460-2075.1991.tb07764.x PMC452918

[B5] van der Hoeven F Zakany J Duboule D Gene transpositions in the *HoxD* complex reveal a hierarchy of regulatory controls Cell 1996 85 1025 1035 10.1016/S0092-8674(00)81303-3 8674109

[B6] Puschel AW Balling R Gruss P Separate elements cause lineage restriction and specify boundaries of Hox-1.1 expression Development 1991 112 279 287 1685116 10.1242/dev.112.1.279

[B7] Gerard M Duboule D Zakany J Structure and activity of regulatory elements involved in the activation of the *Hoxd-11* gene during late gastrulation EMBO J 1993 12 3539 3550 7902810 10.1002/j.1460-2075.1993.tb06028.x PMC413630

[B8] Duboule D Vertebrate *Hox* gene regulation: clustering and/or colinearity? Curr Opin Genet Dev 1998 8 514 518 10.1016/S0959-437X(98)80004-X 9794816

[B9] Maeda RK Karch F The ABC of the BX-C: the bithorax complex explained Development 2006 133 1413 1422 10.1242/dev.02323 16556913

[B10] West AG Gaszner M Felsenfeld G Insulators: many functions, many mechanisms Genes Dev 2002 16 271 288 10.1101/gad.954702 11825869

[B11] Maeda RK Karch F Making connections: boundaries and insulators in *Drosophila* Curr Opin Genet Dev 2007 17 394 399 10.1016/j.gde.2007.08.002 17904351

[B12] Schwartz YB Pirrotta V Polycomb silencing mechanisms and the management of genomic programmes Nat Rev Genet 2007 8 9 22 17173055 10.1038/nrg1981

[B13] Dasari V Mishra RK Epigenetic regulation of genes during development: a conserved theme from flies to mammals J Genet Genomics 2008 35 413 429 10.1016/S1673-8527(08)60059-4 18640621

[B14] Asp P Blum R Vethantham V Parisi F Micsinai M Cheng J Bowman C Kluger Y Dynlacht BD Genome-wide remodeling of the epigenetic landscape during myogenic differentiation Proc Natl Acad Sci USA 2011 108 22 E149 E158 10.1073/pnas.1102223108 21551099 PMC3107312

[B15] Bernstein BE Liu CL Humphrey EL Perlstein EO Schreiber SL Global nucleosome occupancy in yeast Genome Biol 2004 5 R62 10.1186/gb-2004-5-9-r62 15345046 PMC522869

[B16] Yuan GC Liu YJ Dion MF Slack MD Wu LF Altschuler SJ Rando OJ Genome-scale identification of nucleosome positions in S. cerevisiae Science 2005 309 626 630 10.1126/science.1112178 15961632

[B17] Bernstein BE Kamal M Lindblad-Toh K Bekiranov S Bailey DK Huebert DJ McMahon S Karlsson EK Kulbokas EJ 3rd Gingeras TR Schreiber SL Lander ES Genomic maps and comparative analysis of histone modifications in human and mouse Cell 2005 120 169 181 10.1016/j.cell.2005.01.001 15680324

[B18] Schones DE Cui K Cuddapah S Roh TY Barski A Wang Z Wei G Zhao K Dynamic regulation of nucleosome positioning in the human genome Cell 2008 132 887 898 10.1016/j.cell.2008.02.022 18329373 PMC10894452

[B19] Zeltser L Desplan C Heintz N *Hoxb-13*: a new *Hox* gene in a distant region of the *HOXB* cluster maintains colinearity Development 1996 122 2475 2484 8756292 10.1242/dev.122.8.2475

[B20] Chopra VS Cande J Hong JW Levine M Stalled *Hox* promoters as chromosomal boundaries Genes Dev 2009 23 1505 1509 10.1101/gad.1807309 19515973 PMC2704471

[B21] Chung JH Whiteley M Felsenfeld G A 5' element of the chicken β-globin domain serves as an insulator in human erythroid cells and protects against position effect in *Drosophila* Cell 1993 74 505 514 10.1016/0092-8674(93)80052-G 8348617

[B22] Vasanthi D Anant M Srivastava S Mishra RK A functionally conserved boundary element from the mouse *HoxD* locus requires GAGA factor in *Drosophila* Development 2010 137 4239 4247 10.1242/dev.058701 21098566

[B23] Udvardy A Maine E Schedl P The 87A7 chromomere. Identification of novel chromatin structures flanking the heat shock locus that may define the boundaries of higher order domains J Mol Biol 1985 185 341 358 10.1016/0022-2836(85)90408-5 2997449

[B24] Mito Y Henikoff JG Henikoff S Histone replacement marks the boundaries of cis-regulatory domains Science 2007 315 1408 1411 10.1126/science.1134004 17347439

[B25] Dorsett D Distant liaisons: long-range enhancer-promoter interactions in *Drosophila* Curr Opin Genet Dev 1999 9 5 505 514 10.1016/S0959-437X(99)00002-7 10508687

[B26] Chambeyron S Bickmore WA Chromatin decondensation and nuclear reorganization of the *HoxB* locus upon induction of transcription Genes Dev 2004 18 1119 1130 10.1101/gad.292104 15155579 PMC415637

[B27] Berger N Dubreucq B Evolution goes GAGA: GAGA binding proteins across kingdoms Biochim Biophys Acta 2012 1819 863 868 10.1016/j.bbagrm.2012.02.022 22425673

[B28] Farkas G Gausz J Galloni M Reuter G Gyurkovics H Karch F The *Trithorax-like* gene encodes the *Drosophila* GAGA factor Nature 1994 371 806 808 10.1038/371806a0 7935842

[B29] Belozerov VE Majumder P Shen P Cai HN A novel boundary element may facilitate independent gene regulation in the Antennapedia complex of *Drosophila* EMBO J 2003 22 3113 3121 10.1093/emboj/cdg297 12805225 PMC162149

[B30] Schweinsberg S Hagstrom K Gohl D Schedl P Kumar RP Mishra R Karch F The enhancer-blocking activity of the *Fab-7* boundary from the *Drosophila* bithorax complex requires GAGA-factor-binding sites Genetics 2004 168 1371 1384 10.1534/genetics.104.029561 15579691 PMC1448804

[B31] Kim TH Abdullaev ZK Smith AD Ching KA Loukinov DI Green RD Zhang MQ Lobanenkov VV Ren B Analysis of the vertebrate insulator protein CTCF-binding sites in the human genome Cell 2007 128 1231 1245 10.1016/j.cell.2006.12.048 17382889 PMC2572726

[B32] Barski A Cuddapah S Cui K Roh TY Schones DE Wang Z Wei G Chepelev I Zhao K High-resolution profiling of histone methylations in the human genome Cell 2007 129 823 837 10.1016/j.cell.2007.05.009 17512414

[B33] Kim YJ Cecchini KR Kim TH Conserved, developmentally regulated mechanism couples chromosomal looping and heterochromatin barrier activity at the homeobox gene A locus Proc Natl Acad Sci USA 2011 108 7391 7396 10.1073/pnas.1018279108 21502535 PMC3088595

[B34] Soshnikova N Montavon T Leleu M Galjart N Duboule D Functional analysis of CTCF during mammalian limb development Dev Cell 2010 19 819 830 10.1016/j.devcel.2010.11.009 21145498

[B35] Dunn KL Davie JR The many roles of the transcriptional regulator CTCF Biochem Cell Biol 2003 81 161 167 10.1139/o03-052 12897849

[B36] Ferraiuolo MA Rousseau M Miyamoto C Shenker S Wang XQ Nadler M Blanchette M Dostie J The three-dimensional architecture of *Hox* cluster silencing Nucleic Acids Res 2010 38 7472 7484 10.1093/nar/gkq644 20660483 PMC2995065

[B37] Tsukiyama T Becker PB Wu C ATP-dependent nucleosome disruption at a heat-shock promoter mediated by binding of GAGA transcription factor Nature 1994 367 525 532 10.1038/367525a0 8107823

[B38] Nakayama T Shimojima T Hirose S The PBAP remodeling complex is required for histone H3.3 replacement at chromatin boundaries and for boundary functions Development 2012 139 4582 4590 10.1242/dev.083246 23136390

[B39] Yamagishi T Ozawa M Ohtsuka C Ohyama-Goto R Kondo T *Evx2-Hoxd13* intergenic region restricts enhancer association to *Hoxd13* promoter PLoS One 2007 2 e175 10.1371/journal.pone.0000175 17245451 PMC1766471

[B40] Matharu NK Hussain T Sankaranarayanan R Mishra RK Vertebrate homologue of *Drosophila* GAGA factor J Mol Biol 2010 400 434 447 10.1016/j.jmb.2010.05.010 20471984

[B41] Sebastian S Sreenivas P Sambasivan R Cheedipudi S Kandalla P Pavlath GK Dhawan J MLL5, a trithorax homolog, indirectly regulates H3K4 methylation, represses cyclin A2 expression, and promotes myogenic differentiation Proc Natl Acad Sci USA 2009 106 4719 4724 10.1073/pnas.0807136106 19264965 PMC2651835

[B42] Sachidanandan C Sambasivan R Dhawan J Tristetraprolin and LPS-inducible CXC chemokine are rapidly induced in presumptive satellite cells in response to skeletal muscle injury J Cell Sci 2002 115 2701 2712 12077361 10.1242/jcs.115.13.2701

[B43] UCSC genome browserhttp://genome.ucsc.edu/

[B44] GEO databasehttp://www.ncbi.nlm.nih.gov/geo/

[B45] Bailey TL Boden M Buske FA Frith M Grant CE Clementi L Ren J Li WW Noble WS MEME Suite: tools for motif discovery and searching Nucleic Acids Res 2009 37 W202 W208 10.1093/nar/gkp335 19458158 PMC2703892

[B46] Geneious v5.5 2011 http://www.geneious.com

