# Supplementary material for: Vertebrate GAGA factor associated insulator elements demarcate homeotic genes in the HOX clusters
Source: Epigenetics Chromatin. 2013 Apr 22;6:8. doi: 10.1186/1756-8935-6-8 (PMC3639804; doi:10.1186/1756-8935-6-8)
Supplement: Additional file 1: Figure S1 — HFRs across all arrays using histone H3 antibodies. Screenshots from the tiling array data obtained using pan histone H3 (blue track), H3K4me3 (pink track) and H3K27me3 (green track) antibodies, visualized using the UCSC genome browser showing normalized log ratio (NLR) values of probes (y axis) at the Hox clusters (x axis). (A) Large stretches of histone H3 unenriched probes showing negative NLR values across all the arrays are highlighted by boxed regions. Map of all the HFRs (green boxes) further identified by bioinformatics analysis of the probe binding data is presented at the bottom for each cluster. (B) H3K4me3 and H3K27me3 peaks called in the custom tiling arrays show that the HoxB and HoxD clusters are highly enriched for the H3K27me3 mark in G0 cells, as reported previously for proliferating myoblasts and myotubes [14]; genes in the HoxA and HoxC clusters do not share this feature but show some association with H3K4me3. These trends are comparable with those seen in the ChIP-seq data from ENCODE using C2C12 cells (brown tracks). Genomic scale bar is indicated at the top. Figure S2. Map of GAGA motifs and CTCF sites across Hox clusters. Schematic map (drawn to scale) depicting the location of all the GAGA motifs identified by sequence analysis as well as the CTCF sites identified by ChIP-seq in C2C12 cells from the mouse ENCODE project as obtained from the UCSC genome browser (Transcription Factor Binding Sites by ChIP-seq from ENCODE/Caltech). Green boxes indicate HFRs while red bars mark GAGA motifs and grey bars mark CTCF sites. Blue boxes denote the Hox genes with transcriptional orientation; the large intronic regions of Hoxa3 and Hoxd3 genes have been omitted for clarity. Figure S3. DNaseI HS peaks in context of HFRs at the Hox clusters. Schematic map (drawn to scale) depicting the location of all the peaks of DNaseI hypersensitivity identified in skeletal muscle (blue track), mesoderm (black track) and embryonic stem cells (maroon track) from the mou [file 1756-8935-6-8-S1.docx]

**Additional file 1:**

**Figures S1, S2, S3**

**Tables S1-5**

**Figure S1: HFRs across all arrays using histone H3 antibodies**

Screenshots from the tiling array data obtained using pan histone H3 (blue track), H3K4me3 (pink track) and H3K27me3 (green track) antibodies, visualized using the UCSC genome browser showing normalized log ratio (NLR) values of probes (Y-axis) at the Hox clusters (X-axis).

**A**

**
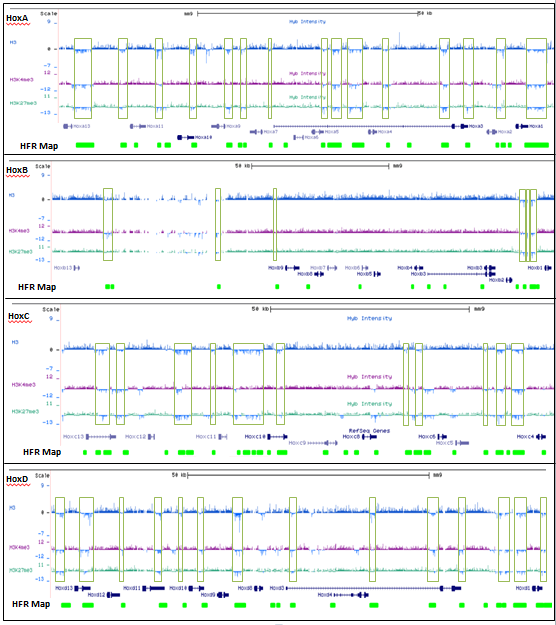
**

**Figure S1A:** Large stretches of histone H3 un-enriched probes showing negative NLR values across all the arrays are highlighted by boxed regions. Map of all the HFRs (green boxes) further identified by bioinformatics analysis of the probe binding data is presented at the bottom for each cluster.

**B**

**
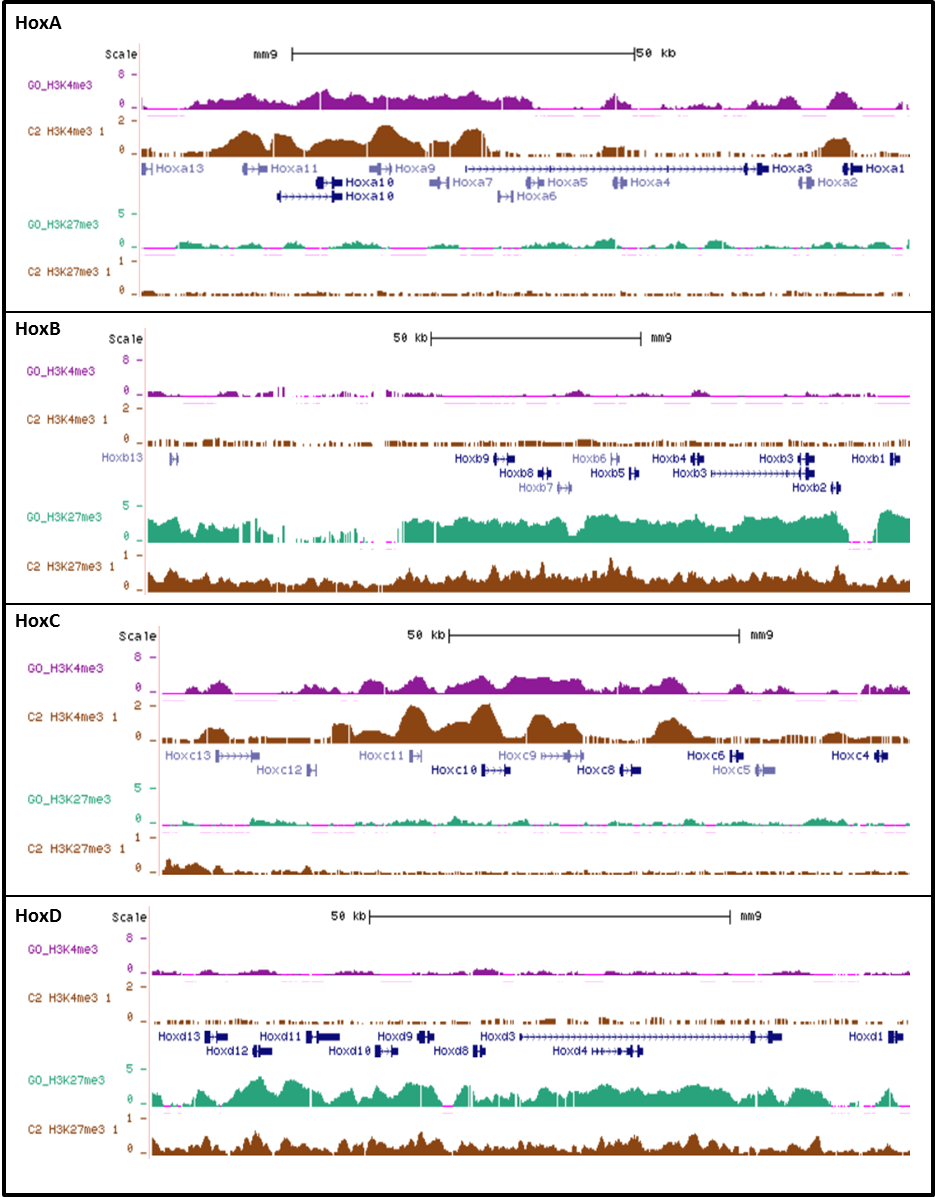
**

**Figure S1B:** H3K4me3 and H3K27me3 peaks identified from the positively enriched probes in the custom tiling arrays show that the HoxB and HoxD clusters are highly enriched for the H3K27me3 mark in G0 cells, as reported previously for proliferating myoblasts and myotubes [46]; genes in the HoxA and HoxC clusters do not share this feature but show some association with H3K4me3. These trends are comparable with those seen in the ChIP-seq data from ENCODE using C2C12 cells (brown tracks). Genomic scale bar is indicated at the top.

**Figure S2: Map of GAGA motifs and CTCF sites across Hox clusters**


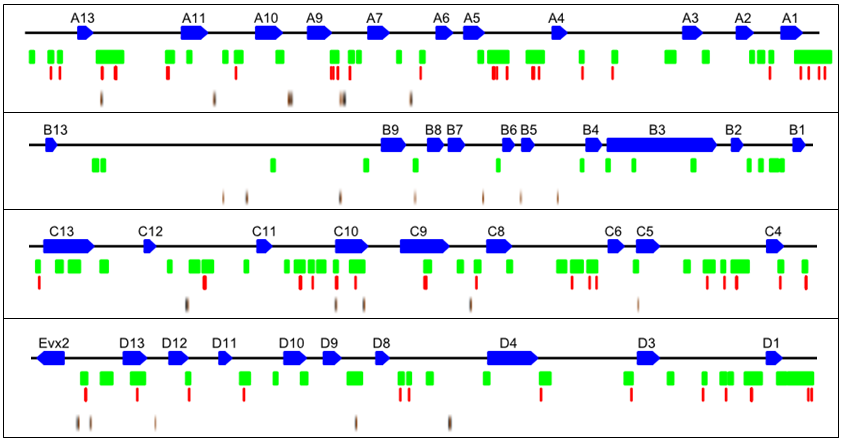


Schematic map (drawn to scale) depicting the location of all the GAGA motifs identified by sequence analysis as well as the CTCF sites identified by ChIP-seq in C2C12 cells from the mouse ENCODE project as obtained from the UCSC genome browser (Transcription Factor Binding Sites by ChIP-seq from ENCODE/Caltech). Green boxes indicate the HFRs while red bars mark GAGA motifs and grey bars mark CTCF sites. Blue boxes denote the Hox genes with transcriptional orientation; the large intronic regions of Hoxa3 and Hoxd3 genes have been omitted for clarity.

**Figure S3: DNaseI HS peaks in context of HFRs at the Hox clusters**


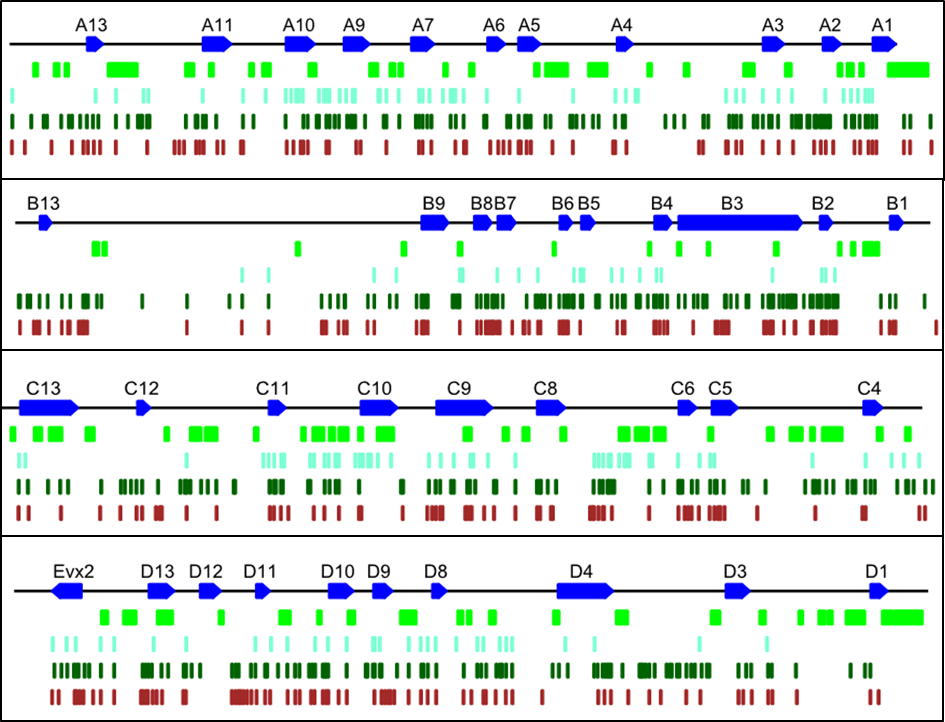


Schematic map (drawn to scale) depicting the location of all the peaks of DNaseI hypersensitivity identified in skeletal muscle (blue track), mesoderm (black track) and embryonic stem cells (maroon track) from the mouse ENCODE project (DNaseI Hypersensitivity by Digital DNaseI from ENCODE/University of Washington). Green boxes indicate the HFRs while blue boxes denote the Hox genes with transcriptional orientation; the large intronic regions of Hoxa3 and Hoxd3 genes have been omitted for clarity.

**Table S1. HFRs across the three arrays**

| **Name** | **H3** | **H3K4me3** | **H3K27me3** |  | **Name** | **H3** | **H3K4me3** | **H3K27me3** |
| --- | --- | --- | --- | --- | --- | --- | --- | --- |
| **HoxA** | | | |  | **HoxC continued** | | | |
| A_DOWN-1.1 | ✓ | ✓ | ✓ |  | C_12-11.1 | ✓ | ✓ | ✓ |
| A_1-2.1 | ✓ | 🗶 | 🗶 |  | C_12-11.2 | ✓ | ✓ | ✓ |
| A_1-2.2 | ✓ | ✓ | ✓ |  | C_12-11.3 | ✓ | ✓ | ✓ |
| A_1-2.3 | ✓ | ✓ | ✓ |  | C_12-11.4 | ✓ | ✓ | ✓ |
| A_2-3.1 | ✓ | ✓ | ✓ |  | C_11-10.1 | ✓ | ✓ | ✓ |
| A_3-4.1 | ✓ | ✓ | ✓ |  | C_11-10.2 | ✓ | 🗶 | 🗶 |
| A_3-4.2 | ✓ | ✓ | ✓ |  | C_11-10.3 | ✓ | ✓ | ✓ |
| A_3-4.3 | ✓ | ✓ | ✓ |  | C_11-10.4 | ✓ | 🗶 | ✓ |
| A_4-5.1 | ✓ | ✓ | ✓ |  | C_11-10.5 | ✓ | 🗶 | 🗶 |
| A_4-5.2 | ✓ | 🗶 | 🗶 |  | C_10.1 | ✓ | 🗶 | ✓ |
| A_5.1 | ✓ | ✓ | ✓ |  | C_9.1 | ✓ | 🗶 | 🗶 |
| A_6-7.1 | ✓ | ✓ | ✓ |  | C_9-8.1 | ✓ | ✓ | ✓ |
| A_6-7.2 | ✓ | ✓ | ✓ |  | C_9-8.2 | ✓ | 🗶 | 🗶 |
| A_7-9.1 | ✓ | 🗶 | ✓ |  | C_8-6.1 | ✓ | ✓ | ✓ |
| A_7-9.2 | ✓ | 🗶 | ✓ |  | C_8-6.2 | ✓ | 🗶 | 🗶 |
| A_7-9.3 | ✓ | ✓ | ✓ |  | C_8-6.3 | ✓ | ✓ | ✓ |
| A_9-10.1 | ✓ | ✓ | ✓ |  | C_8-6.4 | ✓ | ✓ | ✓ |
| A_10-11.1 | ✓ | 🗶 | 🗶 |  | C_6-5.1 | ✓ | ✓ | ✓ |
| A_10-11.2 | ✓ | ✓ | ✓ |  | C_5-4.1 | ✓ | ✓ | ✓ |
| A_11.1 | ✓ | 🗶 | ✓ |  | C_5-4.2 | ✓ | ✓ | ✓ |
| A_11-13.1 | ✓ | ✓ | ✓ |  | C_5-4.3 | ✓ | 🗶 | 🗶 |
| A_11-13.2 | ✓ | ✓ | ✓ |  | C_5-4.4 | ✓ | ✓ | ✓ |
| A.UP.1 | ✓ | ✓ | 🗶 |  | C_4-DOWN.1 | ✓ | 🗶 | ✓ |
| A.UP.2 | ✓ | 🗶 | 🗶 |  | C_DOWN.1 | ✓ | ✓ | ✓ |
| A.UP.3 | ✓ | ✓ | ✓ |  | C_DOWN.2 | ✓ | ✓ | ✓ |
| **HoxB** | | | |  | **HoxD** | | | |
| B_13-9.1 | ✓ | 🗶 | 🗶 |  | D_UP.13 | ✓ | ✓ | ✓ |
| B_13-9.2 | ✓ | ✓ | ✓ |  | D_UP.14 | ✓ | ✓ | ✓ |
| B_13-9.3 | ✓ | ✓ | ✓ |  | D_13.1 | ✓ | ✓ | 🗶 |
| B_13-9.4 | ✓ | ✓ | ✓ |  | D_12-11.1 | ✓ | ✓ | ✓ |
| B_9-8.1 | ✓ | ✓ | ✓ |  | D_11-10.1 | ✓ | ✓ | ✓ |
| B_7-6.1 | ✓ | ✓ | 🗶 |  | D_11-10.2 | ✓ | ✓ | ✓ |
| B_5-4.1 | ✓ | ✓ | ✓ |  | D_10-9.1 | ✓ | ✓ | ✓ |
| B_4-3.1 | ✓ | ✓ | 🗶 |  | D_9-8.1 | ✓ | ✓ | ✓ |
| B_3.1 | ✓ | ✓ | 🗶 |  | D_8-4.1 | ✓ | 🗶 | ✓ |
| B_3.2 | ✓ | 🗶 | 🗶 |  | D_8-4.2 | ✓ | ✓ | 🗶 |
| B_2-1.1 | ✓ | 🗶 | ✓ |  | D_8-4.3 | ✓ | ✓ | ✓ |
| B_2-1.2 | ✓ | ✓ | ✓ |  | D_8-4.4 | ✓ | ✓ | ✓ |
| B_2-1.3 | ✓ | ✓ | ✓ |  | D_4-3.1 | ✓ | 🗶 | 🗶 |
| B_2-1.4 | ✓ | ✓ | ✓ |  | D_4-3.2 | ✓ | ✓ | ✓ |
| **HoxC** | | | |  | D_3-1.1 | ✓ | ✓ | ✓ |
| C_UP.26 | ✓ | ✓ | ✓ |  | D_3-1.2 | ✓ | ✓ | 🗶 |
| C_UP.27 | ✓ | 🗶 | ✓ |  | D_3-1.3 | ✓ | ✓ | ✓ |
| C_UP.28 | ✓ | 🗶 | 🗶 |  | D_3-1.4 | ✓ | ✓ | ✓ |
| C_UP.29 | ✓ | ✓ | 🗶 |  | D_3-1.5 | ✓ | ✓ | 🗶 |
| C_13.1 | ✓ | ✓ | ✓ |  | D_1-DOWN.1 | ✓ | ✓ | ✓ |
| C_13.2 | ✓ | ✓ | ✓ |  | D_DOWN.1 | ✓ | ✓ | ✓ |
| C_13-12.1 | ✓ | ✓ | ✓ |  | D_DOWN.2 | ✓ | ✓ | ✓ |

Presence of the 93 HFRs identified in the pan H3 array was determined across both the modified histone arrays. Tick indicates that >50% of the probes in the region were unenriched (histone free), while cross indicates that they were positively enriched.

**Table S2: List of GAGA factor binding motifs at the Hox clusters**

| **SEQ NAME** | **MOTIF** | **STRAND** | **MOTIF START** | **MOTIF STOP** | **P-VALUE** |
| --- | --- | --- | --- | --- | --- |
| A_10-11.1 | CCCTCTCT | + | 52187883 | 52187890 | 1.55E-05 |
| A_11-13.1 | CCCTCTCT | - | 52197921 | 52197928 | 1.55E-05 |
| A_11-13.1 | CCCTCTCT | + | 52197706 | 52197713 | 1.55E-05 |
| A_11-13.2 | CTCTCCCT | + | 52207402 | 52207409 | 3.79E-05 |
| A_11-13.2 | CCCTCCCT | + | 52207384 | 52207391 | 1.55E-05 |
| A_11-13.2 | CTCTCTCT | + | 52207376 | 52207383 | 3.79E-05 |
| A_11-13.2 | CTCTCTCT | - | 52205482 | 52205489 | 3.79E-05 |
| A_11-13.2 | CTCTCTCT | - | 52205472 | 52205479 | 3.79E-05 |
| A_11-13.2 | CTCTCTCT | - | 52205464 | 52205471 | 3.79E-05 |
| A_11-13.2 | CTCTCTCT | - | 52205456 | 52205463 | 3.79E-05 |
| A_11-13.2 | CTCTCTCT | - | 52205448 | 52205455 | 3.79E-05 |
| A_11-13.2 | CTCTCTCT | - | 52205440 | 52205447 | 3.79E-05 |
| A_11-13.2 | CCCTCCCT | - | 52205428 | 52205435 | 1.55E-05 |
| A_11-13.2 | CCCTCCCT | - | 52205416 | 52205423 | 1.55E-05 |
| A_11-13.2 | CCCTCCCT | - | 52205408 | 52205415 | 1.55E-05 |
| A_11-13.2 | CCCTCCCT | - | 52205400 | 52205407 | 1.55E-05 |
| A_11-13.2 | CCCTCCCT | - | 52205384 | 52205391 | 1.55E-05 |
| A_11-13.2 | CCCTCCCC | - | 52205376 | 52205383 | 4.86E-05 |
| A_11-13.2 | CCCTCCCT | - | 52205351 | 52205358 | 1.55E-05 |
| A_1-2.1 | CCCTCCCC | + | 52110009 | 52110016 | 4.86E-05 |
| A_3-4.2 | CCCTCCCC | + | 52132958 | 52132965 | 4.86E-05 |
| A_3-4.3 | CTCTCCCC | - | 52137345 | 52137352 | 6.41E-05 |
| A_4-5.1 | CCCTCCCT | - | 52144644 | 52144651 | 1.55E-05 |
| A_4-5.1 | CCCTCTCT | - | 52144425 | 52144432 | 1.55E-05 |
| A_4-5.1 | CTCTCTCT | + | 52143680 | 52143687 | 3.79E-05 |
| A_4-5.2 | CCCTCTCC | + | 52150391 | 52150398 | 4.86E-05 |
| A_4-5.2 | CTCTCTCC | + | 52150220 | 52150227 | 6.41E-05 |
| A_4-5.2 | CCCTCTCC | - | 52149822 | 52149829 | 4.86E-05 |
| A_4-5.2 | CCCTCTCT | + | 52148369 | 52148376 | 1.55E-05 |
| A_4-5.2 | CCCTCTCC | - | 52148352 | 52148359 | 4.86E-05 |
| A_6-7.1 | CTCTCTCT | - | 52160950 | 52160957 | 3.79E-05 |
| A_7-9.2 | CTCTCTCT | + | 52171278 | 52171285 | 3.79E-05 |
| A_7-9.2 | CTCTCTCT | + | 52171268 | 52171275 | 3.79E-05 |
| A_7-9.2 | CTCTCTCT | + | 52171260 | 52171267 | 3.79E-05 |
| A_7-9.2 | CTCTCTCT | + | 52171252 | 52171259 | 3.79E-05 |
| A_7-9.2 | CTCTCTCT | + | 52171244 | 52171251 | 3.79E-05 |
| A_7-9.2 | CTCTCTCT | + | 52171236 | 52171243 | 3.79E-05 |
| A_7-9.3 | CCCTCCCT | + | 52174002 | 52174009 | 1.55E-05 |
| A_7-9.3 | CTCTCCCC | + | 52173674 | 52173681 | 6.41E-05 |
| A_7-9.3 | CCCTCCCC | + | 52173039 | 52173046 | 4.86E-05 |
| A_7-9.3 | CTCTCTCT | - | 52172999 | 52173006 | 3.79E-05 |
| A_DOWN-1.1 | CCCTCCCT | + | 52105506 | 52105513 | 1.55E-05 |
| A_DOWN-1.1 | CCCTCCCC | - | 52104340 | 52104347 | 4.86E-05 |
| A_DOWN-1.1 | CCCTCCCT | + | 52102833 | 52102840 | 1.55E-05 |
| A_DOWN-1.1 | CCCTCTCT | + | 52101986 | 52101993 | 1.55E-05 |
| A_UP.1 | CTCTCTCT | - | 52213562 | 52213569 | 3.79E-05 |
| A_UP.2 | CTCTCTCC | - | 52214875 | 52214882 | 6.41E-05 |
| C_10.1 | CTCTCTCT | + | 102800313 | 102800320 | 1.40E-05 |
| C_11-10.2 | CTCTCCCT | + | 102791783 | 102791790 | 2.45E-05 |
| C_11-10.2 | CTCTCTCT | + | 102791695 | 102791702 | 1.40E-05 |
| C_11-10.2 | CTCTCTCT | + | 102791687 | 102791694 | 1.40E-05 |
| C_11-10.2 | CTCTCTCT | + | 102791679 | 102791686 | 1.40E-05 |
| C_11-10.2 | CTCTCTCT | + | 102791671 | 102791678 | 1.40E-05 |
| C_11-10.2 | CTCTCTCT | + | 102791663 | 102791670 | 1.40E-05 |
| C_11-10.2 | CTCTCTCT | + | 102791649 | 102791656 | 1.40E-05 |
| C_11-10.2 | CTCTCCCT | + | 102791637 | 102791644 | 2.45E-05 |
| C_11-10.2 | CTCTCTCT | + | 102791625 | 102791632 | 1.40E-05 |
| C_11-10.3 | CTCTCTCT | + | 102793603 | 102793610 | 1.40E-05 |
| C_11-10.5 | CTCTCTCT | + | 102797441 | 102797448 | 1.40E-05 |
| C_11-10.5 | CTCTCTCT | + | 102797277 | 102797284 | 1.40E-05 |
| C_12-11.3 | CTCTCCCT | - | 102776797 | 102776804 | 2.45E-05 |
| C_12-11.3 | CTCTCCCT | - | 102776781 | 102776788 | 2.45E-05 |
| C_12-11.3 | CTCTCCCT | + | 102776676 | 102776683 | 2.45E-05 |
| C_12-11.3 | CTCTCCCT | - | 102776554 | 102776561 | 2.45E-05 |
| C_12-11.3 | CTCTCCCC | - | 102776544 | 102776551 | 4.29E-05 |
| C_4-DOWN.1 | CTCTCTCC | + | 102866878 | 102866885 | 3.50E-05 |
| C_5-4.2 | CTCTCCCC | + | 102855429 | 102855436 | 4.29E-05 |
| C_5-4.3 | CTCTCCCT | - | 102858128 | 102858135 | 2.45E-05 |
| C_5-4.4 | CTCTCTCT | + | 102860034 | 102860041 | 1.40E-05 |
| C_5-4.4 | CTCTCTCT | + | 102860026 | 102860033 | 1.40E-05 |
| C_5-4.4 | CTCTCTCT | + | 102860018 | 102860025 | 1.40E-05 |
| C_8-6.3 | CTCTCCCC | + | 102834239 | 102834246 | 4.29E-05 |
| C_8-6.4 | CTCTCTCT | + | 102838082 | 102838089 | 1.40E-05 |
| C_8-6.4 | CTCTCTCT | - | 102836998 | 102837005 | 1.40E-05 |
| C_9.1 | CTCTCCCT | + | 102811398 | 102811405 | 2.45E-05 |
| C_9.1 | CTCTCTCC | + | 102811128 | 102811135 | 3.50E-05 |
| C_9.1 | CTCTCCCC | + | 102811343 | 102811350 | 4.29E-05 |
| C_9-8.2 | CTCTCCCC | - | 102819256 | 102819263 | 4.29E-05 |
| C_DOWN.1 | CTCTCTCT | + | 102870927 | 102870934 | 1.40E-05 |
| C_DOWN.1 | CTCTCTCT | + | 102870919 | 102870926 | 1.40E-05 |
| C_DOWN.1 | CTCTCTCT | + | 102870911 | 102870918 | 1.40E-05 |
| C_DOWN.1 | CTCTCTCT | + | 102870903 | 102870910 | 1.40E-05 |
| C_DOWN.1 | CTCTCTCT | + | 102870895 | 102870902 | 1.40E-05 |
| C_DOWN.1 | CTCTCTCT | + | 102870887 | 102870894 | 1.40E-05 |
| C_UP.26 | CTCTCTCT | - | 102742491 | 102742498 | 1.40E-05 |
| C_UP.27 | CTCTCTCT | + | 102745982 | 102745989 | 1.40E-05 |
| C_UP.27 | CTCTCTCT | + | 102745974 | 102745981 | 1.40E-05 |
| C_UP.27 | CTCTCTCT | + | 102745966 | 102745973 | 1.40E-05 |
| C_UP.27 | CTCTCTCT | + | 102745952 | 102745959 | 1.40E-05 |
| C_UP.27 | CTCTCCCT | + | 102746006 | 102746013 | 2.45E-05 |
| C_UP.27 | CTCTCCCT | + | 102745998 | 102746005 | 2.45E-05 |
| C_UP.28 | CTCTCTCC | - | 102747151 | 102747158 | 3.50E-05 |
| C_UP.29 | CTCTCCCC | + | 102750755 | 102750762 | 4.29E-05 |
| D_11-10.1 | AGAAAGAG | + | 74524046 | 74524053 | 5.94E-05 |
| D_12-11.1 | AGGGGGAG | + | 74515984 | 74515991 | 5.94E-05 |
| D_13.1 | AGAGAGAG | - | 74508342 | 74508349 | 2.14E-05 |
| D_DOWN.1 | AGAAGGAG | - | 74607141 | 74607148 | 3.43E-05 |
| D_DOWN.1 | AGGGGGAG | - | 74607633 | 74607640 | 5.94E-05 |
| D_3-1.2 | AGAAAGAG | - | 74591629 | 74591636 | 5.94E-05 |
| D_3-1.3 | AGAAAGAG | + | 74595018 | 74595025 | 5.94E-05 |
| D_3-1.5 | AGAGAGAG | + | 74598802 | 74598809 | 2.14E-05 |
| D_3-1.5 | AGAGAGAG | - | 74598775 | 74598782 | 2.14E-05 |
| D_3-1.5 | AGAGAGAG | - | 74598767 | 74598774 | 2.14E-05 |
| D_3-1.5 | AGAGAGAG | - | 74598759 | 74598766 | 2.14E-05 |
| D_3-1.5 | AGAGAGAG | - | 74598751 | 74598758 | 2.14E-05 |
| D_3-1.5 | AGAGAGAG | - | 74598737 | 74598744 | 2.14E-05 |
| D_4-3.1 | AGGGAGAG | + | 74567774 | 74567781 | 6.79E-05 |
| D_4-3.2 | AGAAAGAG | - | 74581087 | 74581094 | 5.94E-05 |
| D_8-4.1 | AGAGAGAG | - | 74547076 | 74547083 | 2.14E-05 |
| D_8-4.2 | AGAGAGAG | + | 74548357 | 74548364 | 2.14E-05 |
| D_UP.13 | AGAGAGAG | + | 74500767 | 74500774 | 2.14E-05 |
| D_UP.13 | AGAGAGAG | + | 74500759 | 74500766 | 2.14E-05 |
| D_UP.13 | AGAGAGAG | + | 74500745 | 74500752 | 2.14E-05 |

**Table S3.** **Overlap of HFRs with DNaseI HS sites**

| **Cell type** | **HoxA** | **HoxB** | **HoxC** | **HoxD** | **Total** |
| --- | --- | --- | --- | --- | --- |
| **HFRs (this study)** | **25** | **14** | **32** | **22** | **93** |
| **Skeletal Muscle** | 12 | 2 | 14 | 9 | 37 |
| **Mesoderm** | 21 | 10 | 21 | 15 | 67 |
| **ESC** | 15 | 4 | 15 | 13 | 47 |

The number of HFRs overlapping with DNaseI hypersensitive peaks in different tissues as obtained from ENCODE data are tabulated. The total number of HFRs identified in each cluster is indicated in the header.

**Table S4: List of primers for ChIP-qPCR assays**

| **S.No** | **Name** | **Sequence (5'->3')** | **Amplicon (bp)** |
| --- | --- | --- | --- |
| 1 | Mm_HoxA2-3F | GGGAGTCCCTGTTTCAAAGCGCC | 115 |
|  | Mm_HoxA2-3R | TGGCTCCCGAGAGGAAGCAACT |  |
| 2 | Mm_HoxA4F | TGCGGGGCGAACTCCTCGAA | 131 |
|  | Mm_HoxA4R | GCGCAGAAAAACGACACCGCG |  |
| 3 | Mm_HoxA4-5F | ACCCGGCACTCCTGCCTTGG | 150 |
|  | Mm_HoxA4-5R | TCCCCTTCTCTGGCTGGGGTC |  |
| 4 | Mm_HoxA9-10F | ACCACTGGTCCAAGACAGCCACA | 164 |
|  | Mm_HoxA9-10R | TAGCCCGAGCCCCCTGCAAT |  |
| 5 | Mm_HoxA10-11F | ATGCCAACCAGGGGGATGCT | 143 |
|  | Mm_HoxA10-11R | TGCCTAGCAGACTAGCGCAAAG |  |
| 6 | Mm_HoxC8-6F | GGTGGGGTTACAGGAGGCTTGGT | 197 |
|  | Mm_HoxC8-6R | GGCCCCAGCTTTCTGTTTTCTCCT |  |
| 7 | Mm_HoxC8F | TGCCGGTTCCCTCAGAGCGT | 128 |
|  | Mm_HoxC8R | GAGATGCCGGAGGTGCCGTG |  |
| 8 | Mm_HoxC10F | TTCCTCCCGCCCCTCCAACC | 149 |
|  | Mm_HoxC10R | GGCTCCGCGTACGAGTTCGG |  |
| 9 | Mm_HoxC11-10H_F | AGCAGGGGCACTCTGGGCTC | 103 |
|  | Mm_HoxC11-10H_R | AGGGACGCTTTATGGCGGCG |  |
| 10 | Mm_HoxC11-10F | TGGAGACAATGTTCTTTCCCCGGC | 137 |
|  | Mm_HoxC11-10R | CCTGCCCATGTCCTCGGTTTGG |  |
| 11 | Mm_HoxC12-11F | TCCATGAGAGCAAGACAGGGGC | 84 |
|  | Mm_HoxC12-11R | AAGAGGGCTTCATTCTGGGCCT |  |
| 12 | Mm_HoxC13-12F | CCACGTACCGGGCAGGGGAA | 101 |
|  | Mm_HoxC13-12R | GGCATTGGGGTTGTTGAAGAAAGGG |  |
| 13 | Mm_HoxC13F | GGTCCCTTTGCGTCGGCCAA | 83 |
|  | Mm_HoxC13R | TAAAGGGACGGGCAACGCCC |  |
| 14 | Mm_HoxD3-1F | ACCACCAGACACACCTCAACCC | 137 |
|  | Mm_HoxD3-1R | ACGCAACAGACATGAGATGGCCT |  |
| 15 | Mm_HoxD4-3F | TCCTTGGGGTGAGCCTGCCT | 178 |
|  | Mm_HoxD4-3R | CAGCTTCTCAACGTGAGGGCGT |  |
| 16 | Mm_HoxD8F | TGGCGAGGACCCAGACCACT | 84 |
|  | Mm_HoxD8R | AGCGCATCCCAACCTTGTGG |  |
| 17 | Mm_HoxD8-3F | TGGACACCACCGGTCCTCCC | 103 |
|  | Mm_HoxD8-3R | CCCTTTCAAAGCGCAGAAACCTCA |  |
| 18 | Mm_HoxD8-4F | GTGAGAGGCTCCAGCCAGCG | 135 |
|  | Mm_HoxD8-4R | TCAGACCCCCTGCCCCACAA |  |
| 19 | Mm_HoxD9-8F | GGGGACTTGCGGTGTCTGGC | 121 |
|  | Mm_HoxD9-8R | AGTGCAGCAACCATGGGCTCC |  |
| 20 | Mm_HoxD10-9F | GCGCAACCTGTGCTTTTCTGTGC | 128 |
|  | Mm_HoxD10-9R | CCCCAAAGGCTTTCCTCTCCACC |  |
| 21 | Mm_HoxD11-10F | GTGGGCCCAAAAGAGATTTCCGCA | 157 |
|  | Mm_HoxD11-10R | AGGCATGAGCTGCTGTGCATCTT |  |
| 22 | Mm_HoxD12-11F | CCCCACCCCCAACCTTTAGCA | 150 |
|  | Mm_HoxD12-11R | GGTCAGTTGGGGCGAGGCTCA |  |
| 23 | Mm_Evx2-D13F | CCCCATGCCTTCAAAGGGCATCTT | 83 |
|  | Mm_Evx2-D13R | TTCCTGTATGTCACCTCACTCCCAA |  |
| 24 | Control_F | GCTTTCTTGCTACGTGGCTG | 177 |
|  | Control_R | GAGAATGCGAGGGTGAGAAGC |  |

**Table S5: List of primers for cloning test fragments for boundary assays**

| **S.No** | **Region** | **Sequence (5'->3')** | **Amplicon (bp)** |
| --- | --- | --- | --- |
| 1 | A1-2F | GAGCAGCAAGGAGGCCAGCG | 2023 |
|  | A1-2R | ACGCCTTTGACCAGGTGGCT |  |
| 2 | A2-3F | CTGCCGCAGCCGCTGACAG | 733 |
|  | A2-3R | CTGGCCTCTGGCCCTGCAT |  |
| 3 | A3-4F | TGAGGTGATTAATGATTCCCGGGGA | 2030 |
|  | A3-4R | TCCTGCTCCTGTCCTCATCTGCT |  |
| 4 | A3-5F | CCAGTCTTCTGTAGTGTACTTG | 2170 |
|  | A3-5R | CACCTAGGAGGTACTCTGCTGT |  |
| 5 | A4-5F | GTCAAATGGAGTCCAGCGTTAGTGA | 1798 |
|  | A4-5R | CAACCGCTACCTGACCCGCC |  |
| 6 | A6-7F | TGGGGGTACTGCAGCAGGGA | 1076 |
|  | A6-7R | CATTCCCGAGCTGGCTGCCA |  |
| 7 | A9-10F | TGCGAGCCTCTCTGGGGCTC | 1227 |
|  | A9-10R | GTCGGCGCTGAGGTGTCTGG |  |
| 8 | A10-11F | AGCTGGGTCTATGTGAGACACCATT | 1912 |
|  | A10-11R | TCCAGCACTACAGCCCCCTAGAT |  |
| 9 | A13-UPF | AGGGACCTCTGGGGCTGTTTTCT | 1210 |
|  | A13-UPR | GCCTGAGCCTCGCCTTACTGG |  |
| 10 | B2-1F | CGGGCCTTCTCCACTCGTGG | 1248 |
|  | B2-1R | GCCACACTGGGTAAAGTGGTCCC |  |
| 11 | B7-6F | CCCAGAGCCCCTCTGCTGGT | 1422 |
|  | B7-6R | AGCTGCGGGTTTTAAGGGCTGT |  |
| 12 | B9-8F | TCGTGTAAGGCTTAAAACGTGCTGA | 761 |
|  | B9-8R | CCCGCCTGGGGTAGCTTCCT |  |
| 13 | C5-4F | CCGCTCGAGACATGCAGCCCAGCCCCAAC | 1198 |
|  | C5-4R | CCGCTCGAGGCCACTCACGGGCTTGCCTT |  |
| 14 | C8-6F | TCTGGAGGGTCCTAGGCAGACA | 1291 |
|  | C8-6R | GTCCCCTGGGAGCCCCTCAG |  |
| 15 | C9-8R1F | TGGAGCGTGTGGGGAGCACT | 1417 |
|  | C9-8R1R | AAGCAGGGCAGGCTGGAGGA |  |
| 16 | C9-8R2F | AGGCACAGGCCCCAGAGAGG | 1175 |
|  | C9-8R2R | CACTTTCTGGGCGGGGGCAG |  |
| 17 | D3-1F | GGAACCTATTGCACAAAATTGGCGT | 908 |
|  | D3-1R | ATAACCCCTCCCACCCCCAGC |  |
| 18 | D4-3F | GGGAGAGAGTGTGTAGGTAAGGAAGTC | 1370 |
|  | D4-3FR | GCTGGGGTTTTCTTCAGGAAAGCAC |  |
| 19 | D8-3F | ACGATGGTTCAGGTGTCTTCTGGC | 2260 |
|  | D8-3R | TGGCATCAAGGGGTGGGGGT |  |
| 20 | D10-9F | TGATGACTTATGCCTTGTAGCCTTTCC | 349 |
|  | D10-9R | TGAGCTCTCAGGCTGAATCACGA |  |
| 21 | D11-10F | GTGAGGGCTGCAGTGCCCTTT | 1730 |
|  | D11-10R | ACATGCTCAGCTAAGTTGCCAACGC |  |
| 22 | D12-11F | AGTTGTTAGGGGGCTGATGCTCA | 1317 |
|  | D12-11R | GGCCTCGTGGATTGCAGCGT |  |
| 23 | D13-12F | CGCTTCAAGCATCCCGTGTGGT | 747 |
|  | D13-12R | GACCTGTGAGATGAAGTGTGGCCT |  |
| 24 | Evx2-D13F | GGCGCCCCCATCTGTTTTCCT | 1400 |
|  | Evx2-D13R | CCTGCCCGCAGCTCAGACTC |  |
| 26 | A1F | AGCTCCTTGGTCCCACACTTGC | 2109 |
|  | A1R | AGGCTTCTCCCTGGGCTGGG |  |
| 27 | A5F | ACTGCCCTCCCAGTCTGCCC | 966 |
|  | A5R | ACGTCCACGCACTCGCCAC |  |
| 28 | A7F | CGCACAACACTGGCCCAGGG | 1014 |
|  | A7R | GGCTGCTGATGAGGGTCGCC |  |
| 29 | B1F | GCCCTCAGAGGCTGGCTTACG | 541 |
|  | B1R | TGCCTTAGCTGGGACCCGAACA |  |
| 30 | D1F | ACCACCCCCACCATCACCGT | 1200 |
|  | D1R | GAGCCCTGCTGACTGTGGGC |  |
| 31 | D12F | ACAGTGCCCATGCTCCCCCA | 1259 |
|  | D12R | AGGCCAGCAGCTCCCCTCAC |  |
